# Supplementary material for: An Evolutionary Perspective of Dopachrome Tautomerase Enzymes in Metazoans
Source: Genes (Basel). 2019 Jun 28;10(7):495. doi: 10.3390/genes10070495 (PMC6678240; doi:10.3390/genes10070495)
Supplement: Supplementary file 1 [file genes-10-00495-s001.zip › supplementary/Supplementary File 2,3.docx]

**Supplementary File 2.** **Flanking gene and syntenic conservation of DCE/*yellow* genes.**

1. Gene organization of non-insect DCE/*yellow* genes. Phylum, class and species, number of introns, presence of a genomic cluster and flanking genes are reported.

| **Phylum** | **Class** | **Species** | **No. of introns** | **Gene cluster** | **Flanking genes** |
| --- | --- | --- | --- | --- | --- |
| Brachiopoda |  | *L. anatina* | 5,2,2 | Y | (3x) unknown/ANAPC10 |
| Rotifera |  | *A. vaga* | 1 | N | Coproporphyrinogen/tetraspannin |
|  |  |  | 1 | N | Molybdenum cofactor/tetraspannin |
| Mollusca | Cephalopoda | *O. bimaculoides* | 0 | / | -/- |
|  | Bivalvia | *C. gigas* | 0 | / | lancC-like2/transmembrane-channel |
|  |  | *C. virginica* | 0 | / | lancC-like2/transmembrane-channel |
|  |  | *P. fucata* | 0 | / | PKD1L1/ADAMTS17 |
|  |  | *B. platifrons* | 0 | / | acid-sensing ion channel/lancC-like2 |
|  |  | *M. philippinarum* | 1 | N | TLR13/unknown |
|  |  |  | 1 | N | unknown/HES1 |
|  |  | *L. fortunei* | 0  1  3 | N  N  N | -/-  -/ HES1  -/- |
|  |  | *M. yessoensis* | 0-2 | Y | (6x) fibrobl-growth/G-prot receptor |
|  |  |  | 0 | Y | (2x) unknown/unknown |
|  |  |  | 0 | Y | (2x) ovarian abund/serine-threonine |
|  |  |  | 0 | N | (1x) TOM7/serine-threonine |
|  | Gastropoda | *H. discus hannai* | 0 | Y | (3x) -/- |
|  |  |  | 0 | N | (1x) rho-GTPase/Zn-finger MYM |
|  |  |  | 1 | N | (1x) unknown/- |
|  |  |  | 0 | N | (1x) -/polycystin-2 |
| Crustacea | Maxillopoda | *T. kingsejongensis* | 3  4 | N  N | G-nucleotide binding protein/-  rdgA/unknown |
| Chordata | / | *B. floridae* | 7 | N | laccase/sulfotransferase |
|  |  |  | 6 | N | laccase/sulfotransferase |
|  |  | *B. belcheri* | 6 | N | N-acyltransferase/ABC transporter |
|  |  |  | 6 | N | MFHAS1 /FAM124Alike |

1. Sinthenic conservation of Ostreoidae DCE/*yellow* genes. MAUVE alignment of (A) *C. virginica*, (B) *S. glomerata* and *C. gigas* genome regions referring to the DCE/*yellow* locus (+/- 35 kb). (D) gene annotation. The DCE/*yellow* gene regions are included in the red box.


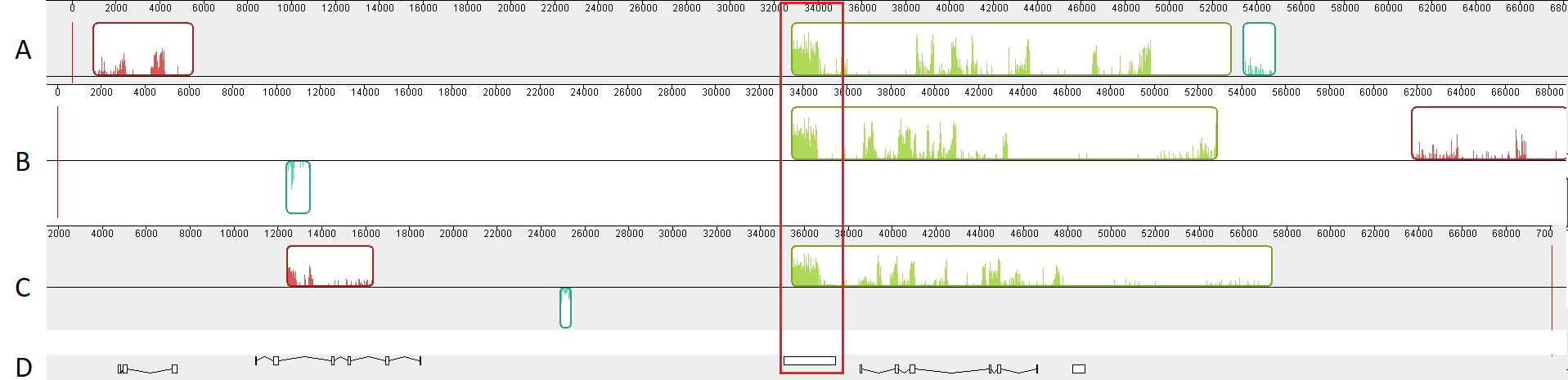


**Supplementary File 3.** **Validation of the two-domain *C. gigas* DCE/*yellow* gene.**

The unique DCE/*yellow* gene identified in the *C. gigas* genome (EKC25673) is intronless and encoded a two-MRJP domains protein. To support the structure of this gene, we sequenced the RNA of two oyster gill samples collected from the lagoon of Goro (Italy) and from a control tank, obtaining 175 million reads (Supplementary File 2). A total of 13,994 reads mapped on the DCE/*yellow* genome locus, showing a continuous coverage distribution in support of the absence of an intron (Figure A). *De-novo* assemblies performed separately on the two RNA-seq datasets allowed us to identify two identical contigs including the full-length DCE/*yellow* transcript (3,149 nt, deposited at NCBI with ID MH719081). This mRNA sequence included a 2,385-nucleotide long ORF, corresponding to a 795 aa protein (Figure B). The Italian DCE/*yellow* variant included 33 nucleotidic differences, corresponding to 13 aminoacidic substitutions when compared with the genome reference.


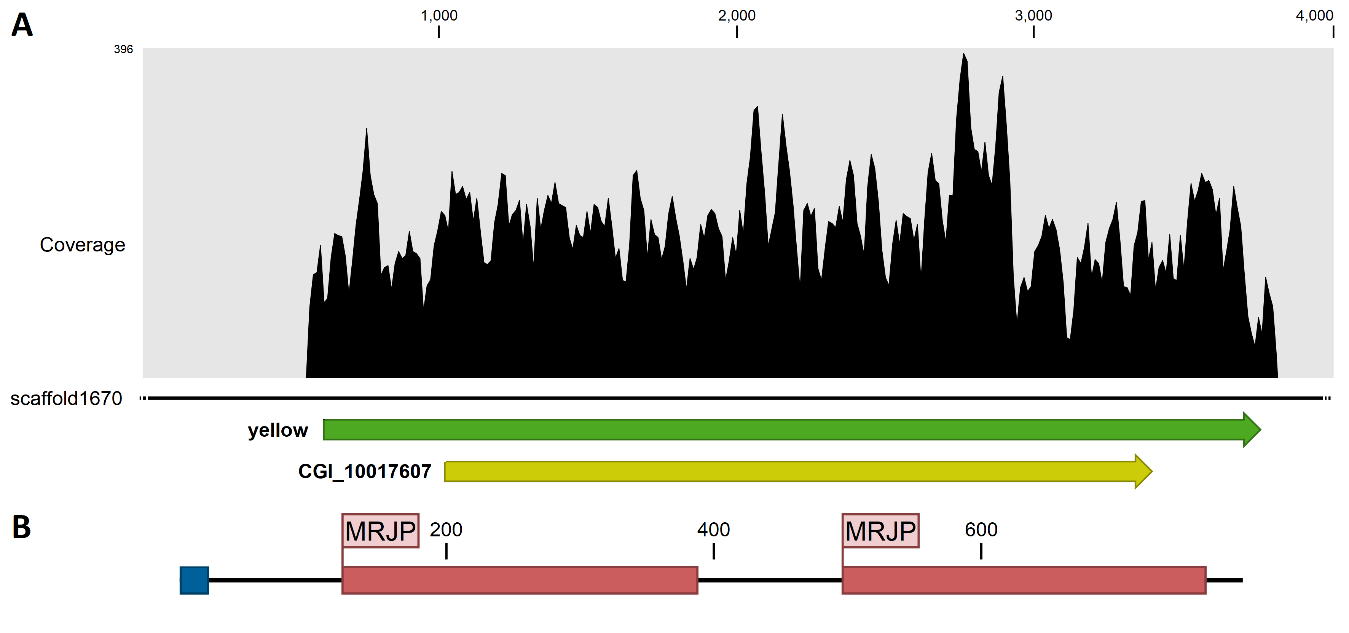


**Figure.** *C. gigas* DCE/*yellow* gene. **A.** RNA-seq coverage graph of the yellow locus on the genome scaffold1670, the whole mRNA annotation (green arrow) and the available gene annotation limited to the CDS only (yellow arrow) were reported. **B.** DCE/*yellow* protein layout, the signal peptide region (blue) and the two MRJP domains were reported.
